# 1105. Population Pharmacokinetic Analyses for Tebipenem After the Administration of Tebipenem Pivoxil Hydrobromide

**DOI:** 10.1093/ofid/ofab466.1299

**Published:** 2021-12-04

**Authors:** Harish Ganesan, Vipul Kumar, Sujata M Bhavnani, David Melnick, Christopher M Rubino

**Affiliations:** 1 Institute for Clinical Pharmacodynamics, Schenectady, New York; 2 Spero Therapeutics, Cambridge, Massachusetts; 3 Institute for Clinical Pharmacodynamics, Inc., Schenectady, NY

## Abstract

**Background:**

Tebipenem pivoxil hydrobromide (TBP-PI-HBr) is an oral prodrug that is converted to tebipenem (TBP), the active moiety. TBP is a carbapenem with activity against multidrug-resistant Gram-negative pathogens, including extended-spectrum-β-lactamase-producing Enterobacterales and is being developed for treating complicated urinary tract infections (cUTI) and acute pyelonephritis (AP). Data from three Phase 1 studies and one Phase 3 study in patients with cUTI/AP were used to develop a population pharmacokinetic (PPK) model for TBP and identify covariates that described the variability in TBP pharmacokinetics (PK).

**Methods:**

The PPK model was developed using TBP plasma and urine concentration-time data from the above-described Phase 1 and 3 studies. TBPPI-HBr doses, which ranged from 100 to 900 mg, were administered as single or multiple doses every 8 hours. After development of the structural model, stepwise forward and backward selection procedures were used to identify significant covariate relationships. The robustness of the final PPK model was assessed using a prediction-corrected visual predictive check (PC-VPC).

**Results:**

The final dataset included 3448 plasma concentrations from 99 Phase 1 subjects and 647 Phase 3 patients and urine concentrations from 128 Phase 1 subjects. A two-compartment model with linear, first-order elimination and transit compartments to describe the rate of drug absorption after oral administration of TBP-PI-HBr best described TBP PK. The most clinically significant covariate effect, which would warrant dose adjustment, was the relationship between apparent oral clearance and creatinine clearance. In contrast, age, body size, sex, and fed status each had a minimal impact on TBP exposure. The PC-VPC showed good agreement between median simulated plasma concentrations based on the final PPK model and the median observed plasma concentrations for the pooled dataset (Figure 1).

Figure 1. Prediction-corrected visual predictive check plot for the final population PK model using the pooled analysis dataset

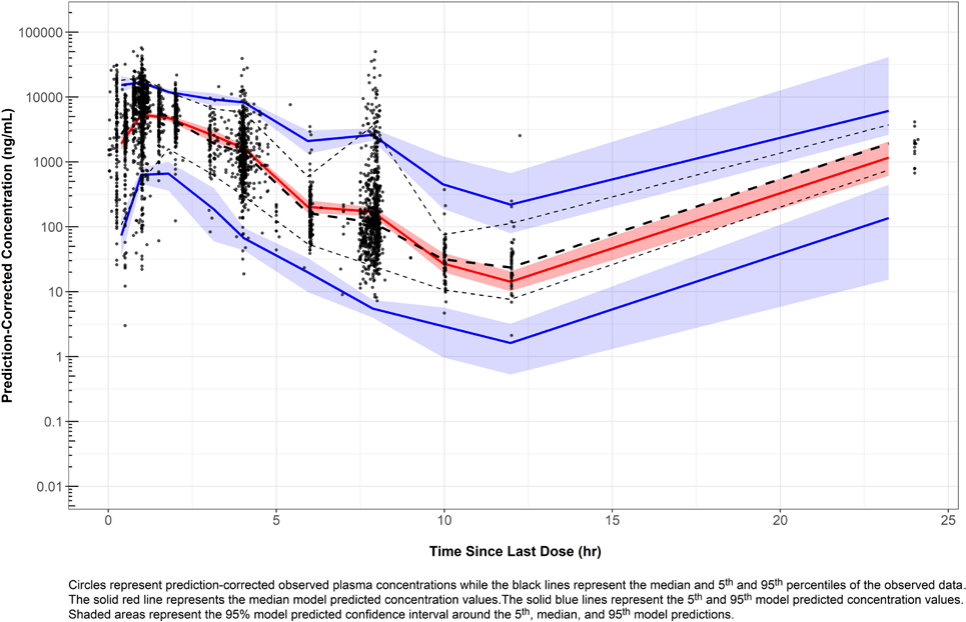

**Conclusion:**

A robust description of TBP plasma PK in subjects and patients with cUTI/AP was achieved, such that derived measures of TBP plasma exposure are expected to be both accurate and precise. The population PK model was considered appropriate for model-based simulations and the assessment of PK-PD relationships for TBP.

**Disclosures:**

**Harish Ganesan, M.S.**, **3-V Biosciences** (Grant/Research Support)**Achogen** (Grant/Research Support)**Amplyx Pharmaceuticals, Inc.** (Grant/Research Support)**Arixa Pharmaceuticals** (Grant/Research Support)**Arsanis Inc.** (Grant/Research Support)**B. Braun Medical Inc.** (Grant/Research Support)**Basilea Pharmaceutica** (Grant/Research Support)**BLC USA** (Grant/Research Support)**Boston Pharmaceuticals** (Grant/Research Support)**Bravos Biosciences, LLC** (Grant/Research Support)**Cidara Therapeutics Inc.** (Grant/Research Support)**Cipla, USA** (Grant/Research Support)**Corcept Therapeutics** (Grant/Research Support)**Cumberland Pharmaceuticals** (Grant/Research Support)**Debiopharm International SA** (Grant/Research Support)**Discuva Limited** (Grant/Research Support)**Emerald Lake Technologies** (Grant/Research Support)**Enhanced Pharmacodynamics** (Grant/Research Support)**Entasis Therapeutics** (Grant/Research Support)**E-Scape Bio** (Grant/Research Support)**Genentech** (Grant/Research Support)**Geom Therapeutics, Inc.** (Grant/Research Support)**GlaxoSmithKline** (Grant/Research Support)**Hoffmann-La Roche** (Grant/Research Support)**Horizon Orphan LLC** (Grant/Research Support)**ICPD Biosciences, LLC** (Grant/Research Support)**Indalo Therapeutics** (Grant/Research Support)**Insmed Inc.** (Grant/Research Support)**Institute for Clinical Pharmacodynamics** (Employee)**Iterum** (Grant/Research Support)**KBP Biosciences USA** (Grant/Research Support)**Kyoto Biopharma, Inc.** (Grant/Research Support)**Matinas** (Grant/Research Support)**Meiji Seika Pharma Co., Ltd.** (Grant/Research Support)**Melinta Therapeutics** (Grant/Research Support)**Menarini Ricerche S.p.A.** (Grant/Research Support)**Merck & Co., Inc** (Grant/Research Support)**Mutabilis** (Grant/Research Support)**Nabriva Therapeutics AG** (Grant/Research Support)**Naeja-RGM Pharmaceuticals** (Grant/Research Support)**Nosopharm SAS** (Grant/Research Support)**Novartis Pharmaceuticals Corp.** (Grant/Research Support)**NuCana Biomed** (Grant/Research Support)**Paratek Pharmaceuticals, Inc.** (Grant/Research Support)**Polyphor, Ltd.** (Grant/Research Support)**Prothena Corporation** (Grant/Research Support)**PTC Therapeutics** (Grant/Research Support)**Rempex Pharmaceuticals** (Grant/Research Support)**Roche TCRC** (Grant/Research Support)**Sagimet** (Grant/Research Support)**scPharmaceuticals Inc.** (Grant/Research Support)**Scynexis** (Grant/Research Support)**Spero Therapeutics** (Grant/Research Support)**TauRx Therapeutics** (Grant/Research Support)**Tetraphase Pharmaceuticals** (Grant/Research Support)**Theravance Biopharma Pharmaceutica** (Grant/Research Support)**USCAST** (Grant/Research Support)**VenatoRx** (Grant/Research Support)**Vical Incorporated** (Grant/Research Support)**Wockhardt Bio AG** (Grant/Research Support)**Zavante Therapeutics** (Grant/Research Support)**Zogenix International** (Grant/Research Support) **Vipul Kumar, PhD**, **Spero Therapeutics** (Employee, Shareholder) **Sujata M. Bhavnani, Pharm.D., M.S., FIDSA**, **3-V Biosciences** (Grant/Research Support)**Achogen** (Grant/Research Support)**Amplyx Pharmaceuticals, Inc.** (Grant/Research Support)**Arixa Pharmaceuticals** (Grant/Research Support)**Arsanis Inc.** (Grant/Research Support)**B. Braun Medical Inc.** (Grant/Research Support)**Basilea Pharmaceutica** (Grant/Research Support)**BLC USA** (Grant/Research Support)**Boston Pharmaceuticals** (Grant/Research Support)**Bravos Biosciences, LLC** (Grant/Research Support, Other Financial or Material Support, member/owner)**Cidara Therapeutics Inc.** (Grant/Research Support)**Cipla, USA** (Grant/Research Support)**Corcept Therapeutics** (Grant/Research Support)**Cumberland Pharmaceuticals** (Grant/Research Support)**Debiopharm International SA** (Grant/Research Support)**Discuva Limited** (Grant/Research Support)**Emerald Lake Technologies** (Grant/Research Support)**Enhanced Pharmacodynamics** (Grant/Research Support)**Entasis Therapeutics** (Grant/Research Support)**E-Scape Bio** (Grant/Research Support)**Genentech** (Grant/Research Support)**Geom Therapeutics, Inc.** (Grant/Research Support)**GlaxoSmithKline** (Grant/Research Support)**Hoffmann-La Roche** (Grant/Research Support)**Horizon Orphan LLC** (Grant/Research Support)**ICPD Biosciences, LLC** (Grant/Research Support, Other Financial or Material Support, member/owner)**Indalo Therapeutics** (Grant/Research Support)**Insmed Inc.** (Grant/Research Support)**Institute for Clinical Pharmacodynamics** (Employee)**Iterum** (Grant/Research Support)**KBP Biosciences USA** (Grant/Research Support)**Kyoto Biopharma, Inc.** (Grant/Research Support)**Matinas** (Grant/Research Support)**Meiji Seika Pharma Co., Ltd.** (Grant/Research Support)**Melinta Therapeutics** (Grant/Research Support)**Menarini Ricerche S.p.A.** (Grant/Research Support)**Merck & Co., Inc** (Grant/Research Support)**Mutabilis** (Grant/Research Support)**Nabriva Therapeutics AG** (Grant/Research Support)**Naeja-RGM Pharmaceuticals** (Grant/Research Support)**Nosopharm SAS** (Grant/Research Support)**Novartis Pharmaceuticals Corp.** (Grant/Research Support)**NuCana Biomed** (Grant/Research Support)**Paratek Pharmaceuticals, Inc.** (Grant/Research Support)**Polyphor, Ltd.** (Grant/Research Support)**Prothena Corporation** (Grant/Research Support)**PTC Therapeutics** (Grant/Research Support)**Rempex Pharmaceuticals** (Grant/Research Support)**Roche TCRC** (Grant/Research Support)**Sagimet** (Grant/Research Support)**scPharmaceuticals Inc.** (Grant/Research Support)**Scynexis** (Grant/Research Support)**Spero Therapeutics** (Grant/Research Support)**TauRx Therapeutics** (Grant/Research Support)**Tetraphase Pharmaceuticals** (Grant/Research Support)**Theravance Biopharma Pharmaceutica** (Grant/Research Support)**USCAST** (Grant/Research Support)**VenatoRx** (Grant/Research Support)**Vical Incorporated** (Grant/Research Support)**Wockhardt Bio AG** (Grant/Research Support)**Zavante Therapeutics** (Grant/Research Support)**Zogenix International** (Grant/Research Support) **David Melnick, MD**, **Spero Therapeutics** (Employee, Shareholder) **Christopher M. Rubino, Pharm.D.**, **3-V Biosciences** (Grant/Research Support)**Achogen** (Grant/Research Support)**Amplyx Pharmaceuticals, Inc.** (Grant/Research Support)**Arixa Pharmaceuticals** (Grant/Research Support)**Arsanis Inc.** (Grant/Research Support)**B. Braun Medical Inc.** (Grant/Research Support)**Basilea Pharmaceutica** (Grant/Research Support)**BLC USA** (Research Grant or Support)**Boston Pharmaceuticals** (Grant/Research Support)**Bravos Biosciences, LLC** (Grant/Research Support, Other Financial or Material Support, member/owner)**Cidara Therapeutics Inc.** (Grant/Research Support)**Cipla, USA** (Grant/Research Support)**Corcept Therapeutics** (Grant/Research Support)**Cumberland Pharmaceuticals** (Grant/Research Support)**Debiopharm International SA** (Grant/Research Support)**Discuva Limited** (Grant/Research Support)**Emerald Lake Technologies** (Grant/Research Support)**Enhanced Pharmacodynamics** (Grant/Research Support)**Entasis Therapeutics** (Grant/Research Support)**E-Scape Bio** (Grant/Research Support)**Genentech** (Grant/Research Support)**Geom Therapeutics, Inc.** (Grant/Research Support)**GlaxoSmithKline** (Grant/Research Support)**Hoffmann-La Roche** (Grant/Research Support)**Horizon Orphan LLC** (Grant/Research Support)**ICPD Biosciences, LLC** (Grant/Research Support, Other Financial or Material Support, member/owner)**Indalo Therapeutics** (Grant/Research Support)**Insmed Inc.** (Grant/Research Support)**Institute for Clinical Pharmacodynamics** (Employee)**Iterum** (Grant/Research Support)**KBP Biosciences USA** (Grant/Research Support)**Kyoto Biopharma, Inc.** (Grant/Research Support)**Matinas** (Grant/Research Support)**Meiji Seika Pharma Co., Ltd.** (Grant/Research Support)**Melinta Therapeutics** (Grant/Research Support)**Menarini Ricerche S.p.A.** (Grant/Research Support)**Merck & Co., Inc** (Grant/Research Support)**Mutabilis** (Grant/Research Support)**Nabriva Therapeutics AG** (Grant/Research Support)**Naeja-RGM Pharmaceuticals** (Grant/Research Support)**Nosopharm SAS** (Grant/Research Support)**Novartis Pharmaceuticals Corp.** (Grant/Research Support)**NuCana Biomed** (Grant/Research Support)**Paratek Pharmaceuticals, Inc.** (Grant/Research Support)**Polyphor, Ltd.** (Grant/Research Support)**Prothena Corporation** (Grant/Research Support)**PTC Therapeutics** (Grant/Research Support)**Rempex Pharmaceuticals** (Grant/Research Support)**Roche TCRC** (Grant/Research Support)**Sagimet** (Grant/Research Support)**scPharmaceuticals Inc.** (Grant/Research Support)**Scynexis** (Grant/Research Support)**Spero Therapeutics** (Grant/Research Support)**TauRx Therapeutics** (Grant/Research Support)**Tetraphase Pharmaceuticals** (Grant/Research Support)**Theravance Biopharma Pharmaceutica** (Grant/Research Support)**USCAST** (Grant/Research Support)**VenatoRx** (Grant/Research Support)**Vical Incorporated** (Grant/Research Support)**Wockhardt Bio AG** (Grant/Research Support)**Zavante Therapeutics** (Grant/Research Support)**Zogenix International** (Grant/Research Support)

